# Structural Disorder and Collective Behavior of Two-Dimensional Magnetic Nanostructures

**DOI:** 10.3390/nano11061392

**Published:** 2021-05-25

**Authors:** David Gallina, G. M. Pastor

**Affiliations:** Institut für Theoretische Physik, Universität Kassel, Heinrich-Plett-Straße 40, 34132 Kassel, Germany; pastor@uni-kassel.de

**Keywords:** nanomagnetism, disordered systems, magnetic order, dipolar interactions, theoretical models

## Abstract

Structural disorder has been shown to be responsible for profound changes of the interaction-energy landscapes and collective dynamics of two-dimensional (2D) magnetic nanostructures. Weakly-disordered 2D ensembles have a few particularly stable magnetic configurations with large basins of attraction from which the higher-energy metastable configurations are separated by only small downward barriers. In contrast, strongly-disordered ensembles have rough energy landscapes with a large number of low-energy local minima separated by relatively large energy barriers. Consequently, the former show good-structure-seeker behavior with an unhindered relaxation dynamics that is funnelled towards the global minimum, whereas the latter show a time evolution involving multiple time scales and trapping which is reminiscent of glasses. Although these general trends have been clearly established, a detailed assessment of the extent of these effects in specific nanostructure realizations remains elusive. The present study quantifies the disorder-induced changes in the interaction-energy landscape of two-dimensional dipole-coupled magnetic nanoparticles as a function of the magnetic configuration of the ensembles. Representative examples of weakly-disordered square-lattice arrangements, showing good structure-seeker behavior, and of strongly-disordered arrangements, showing spin-glass-like behavior, are considered. The topology of the kinetic networks of metastable magnetic configurations is analyzed. The consequences of disorder on the morphology of the interaction-energy landscapes are revealed by contrasting the corresponding disconnectivity graphs. The correlations between the characteristics of the energy landscapes and the Markovian dynamics of the various magnetic nanostructures are quantified by calculating the field-free relaxation time evolution after either magnetic saturation or thermal quenching and by comparing them with the corresponding averages over a large number of structural arrangements. Common trends and system-specific features are identified and discussed.

## 1. Introduction

Two-dimensional (2D) magnetic nanoparticle ensembles have been the focus of a wide range of experimental and theoretical research activity in past years. They constitute a most challenging fundamental research object, in which reduced dimensionality, competing interactions and disorder merge resulting in novel collective behaviors [1,2,3,4,5,6,7,8,9]. A common feature of magnetic nanostructures is the increasing relevance of uncertainties and potential defects in the manufacturing process. For instance, different fabrication processes can yield very different structural arrangements of the magnetic nanoparticles (MNPs) ranging from well-defined long-range order, as in many lithographic samples and auto-organized materials, to highly-disordered samples, as in materials obtained from cluster-beam deposition [10,11,12,13]. It is the main goal of this paper to quantify how the different structural arrangements affect the properties of MNP ensembles.

The magnetic properties of 2D nanostructures are known to depend not only on the size and composition of the nanoparticles (NPs), regarded as individual finite-size objects, but also on the filling factor, surface coverage and geometrical arrangement of the NP ensembles made out of them [14,15,16,17,18,19,20,21]. From the single-particle perspective, a large number of theoretical and experimental studies have revealed the strong size, structural and composition dependence of cluster magnetism (see, for instance, Refs. [22,23,24,25,26,27,28,29,30,31,32,33,34,35,36,37] and references therein). A microscopic understanding of the magnetic behavior of these nano-objects is certainly important not only from a fundamental perspective, but also in view of any knowledge-based nanomaterial development. Nevertheless, as far as the collective behavior of NP ensembles is concerned, the details of the intrinsic cluster properties are not so important, provided that the NPs have a non-vanishing net magnetization whose orientation entirely defines the low-energy NP state. Therefore, the present paper pays particular attention to the interactions among the NPs. Indeed, from this perspective, different coupling regimes must be distinguished. In weakly-interacting 2D ensembles, the surface coverage is low and the magnetic properties are dominated by local contributions such as the magnetization and magnetic anisotropy of the particles themselves. Consequently, the dynamics of NP ensembles is governed by local reorientations of the magnetic moments of individual nanoparticles. The details of the structural arrangement are not important from a qualitative perspective. A far more complex and challenging situation is found in the strongly-interacting 2D ensembles to be considered in the present study, since the surface coverage is high and the underlying structure plays an important role [14,15,16,17,18,19,20,21]. In the strong-coupling regime, any change in the orientation of the magnetization of only one particle inevitably induces changes in the magnetization directions of the neighboring particles. Therefore, the single-particle viewpoint is no longer meaningful. The cooperative many-body character of the ensemble conditions the overall magnetic response. As a result, even the most basic elementary transitions, such as fluctuations between two nearby metastable states, involve simultaneous and collective changes of the magnetization directions of an important number of NPs [19,20,21]. Thus, the many-body behavior of the whole nanostructures must be considered from the very beginning, which renders the physical problem extremely exciting and challenging.

Previous experimental and numerical studies of strongly-interacting 2D ensembles of magnetic nanoparticles have revealed a variety of fascinating physical phenomena, including long-range-order phase transitions, continuous ground-state degeneracies, and order-by-disorder effects [14,15,16,17,18]. In addition, remarkable non-equilibrium phenomena have been observed such as dynamical slowing down, ergodicity breaking, memory effects, and aging [38,39,40,41,42,43], More recently, it has been shown that many of these effects can be understood as the consequence of profound qualitative changes in the interaction-energy landscapes of these systems which are associated to the disordered structural arrangement of the MNPs [44]. On the one hand, ensembles with a high point-group symmetry and a small degree of structural disorder, such as weakly-disordered square and triangular ensembles, have good structure-seeking energy landscapes with a clear global minimum, long-range order and fast unhindered relaxation dynamics. On the other hand, strongly-disordered ensembles show very rough and frustrated energy landscapes with a high number of low-energy local minima separated by large energy barriers resulting and a glass-like relaxation dynamics [44]. Hence, the structural arrangement and its disorder, which is at least to some extent unavoidable in experiment, play a central role in the equilibrium and non-equilibrium properties of these systems. Achieving a detailed microscopic understanding of the correlation between the degree of structural disorder and the nanostructure magnetic behavior is of utmost importance.

The intrinsic randomness of disordered systems implies a number of regularities mostly related to the self averaging proper to macroscopic systems, which call for a statistical description, and yet the statistical perspective alone does not provide sufficient insight on the microscopic origin of a macroscopic behavior, for example, on the nature of the relaxation dynamics. This is even more so in complex systems like those considered in this paper, which involve many coupled degrees of freedom leading to a multitude of metastable states, which are interconnected through an intricate network of elementary transitions. It is therefore of considerable interest to understand how disorder affects the energy landscapes of magnetic NP ensembles, not only by considering different lattice structures with different degrees of disorder, but also by contrasting different concrete realizations of disordered nanostructures corresponding to the same global criteria (i.e., particle size, lattice geometry, coverage, degree of disorder, etc.). It is the purpose of the present contribution to report on the results of such investigations. In this way, both general trends as well as the extent of disorder-induced fluctuations in the interaction-energy landscapes are identified.

The remainder of the paper is then organized as follows: in Section 2 the theoretical background is presented by specifying the considered nanostructure model as well as the different methods used for characterizing the energy landscapes. In Section 3 representative realizations of weakly-disordered and strongly-disordered ensembles are investigated and discussed in some detail by contrasting their kinetic networks, disconnectivity graphs and Markovian relaxation dynamics. The paper is closed in Section 4 by summarizing the main conclusions and by pointing out some relevant extensions and implications of this study.

## 2. Methods and Theoretical Background

### 2.1. Nanostructure Model

The two-dimensional ensembles of strongly interacting magnetic nanoparticles are described by considering *N* non-overlapping spherical particles, which are contained in a square unit cell with periodic boundary conditions. Since the particles are spherical-like, their size is small, in the range of 5–10 nm, and the ferromagnetic exchange couplings between the local atomic magnetic moments are strong, the particles can be treated as superspins. Each particle *k* can be described by a single classical magnetic moment ${\vec{m}}_{k}$ with a fixed module [19,20,21,44]. Since the orientation of ${\vec{m}}_{k}$ is defined by the polar and azimuthal angles $\theta_{k}$ and $\varphi_{k}$, the magnetic configuration of the whole nanostructure is characterized by the set of *N* pairs of angles $\left\{\theta_{k},\varphi_{k}\right\}$ with k=1,…,N. In this context, it is important to recall that magnetism in thermal equilibrium can have very different quantum origins. Consequently, depending on the considered material, different size and structural dependent cluster properties are observed. Nevertheless, the present description of the interaction energy of NP ensembles applies regardless of the intrinsic cluster details, as long as the NPs are small enough to be regarded as a single magnetic domain and the NP magnetic state is characterized by the orientations of the NP magnetizations. In the following calculations we consider Fe particles having a diameter of ϕ=3nm, which are known to be ferromagnetic at low temperatures. The NP magnetization is assumed for simplicity to be bulk-like, which results in a total NP magnetic moment $|\vec{m}|=2.55\times 10^{3}\mu_{B}$. Further details on the structural arrangement of NPs in the 2D ensembles are given in Section 3.

The spherical-like shape of the MNPs implies that the magnetic anisotropies are weak and that higher order multipole corrections can be neglected for simplicity. Hence, we focus on the dipole interactions. For any locations ${\vec{r}}_{k}$ and magnetic moments ${\vec{m}}_{k}$ of the NPs, the total dipolar energy of the system is given by

(1) $$E=\frac{\mu_{0}}{8\pi }\sum_{k\neq l}\left[\frac{{\vec{m}}_{k}\cdot {\vec{m}}_{l}}{r_{kl}^{3}}-\frac{3\left({\vec{m}}_{k}\cdot {\vec{r}}_{kl}\right)\left({\vec{m}}_{l}\cdot {\vec{r}}_{kl}\right)}{r_{kl}^{5}}\right],$$

where ${\vec{r}}_{kl}={\vec{r}}_{k}-{\vec{r}}_{l}$ is the vector connecting the centers of particles *k* and *l*, $r_{kl}=\left|{\vec{r}}_{kl}\right|$ the corresponding distance, and $\mu_{0}$ the vacuum permeability. Extending the present classical-energy expression to include other contributions phenomenologically (e.g., local magnetic anisotropies, higher multipole moments, superexchange or Ruderman–Kittel–Kasuya–Yoshida interactions, etc.) is straightforward.

In order to investigate the effects of structural disorder on the collective behavior of ensembles of magnetic nanoparticles, we consider two different types of geometrical arrangements of the MNPs: (i) weakly-disordered square-lattice structures and (ii) strongly-disordered structures. The weakly-disordered square structures are created by displacing the particles from the sites of a perfectly periodic square lattice according to a Gaussian distribution with zero mean and standard deviation $\sigma_{r}$. The strongly-disordered ensembles are created by randomly placing the non-overlapping particles in the unit cell until the desired surface coverage is reached. Illustrations of representative weakly-disordered and strongly-disordered ensembles can be found in Section 3.

### 2.2. Energy Landscapes

The equilibrium and dynamic properties of classical systems are ultimately governed by the underlying energy as a function of the relevant degrees of freedom. In the present case, these are the azimuthal and polar angles $\left\{\theta_{k},\varphi_{k}\right\}$ which describe the orientation of the magnetic moments ${\vec{m}}_{k}$ of all MNPs. A meaningful simplification of the energy landscape (EL) $E(\theta_{1},\varphi_{1},\ldots ,\theta_{N},\varphi_{N})$ can be achieved by identifying its stationary states, namely, its local minima (LM) and transition states (TS) [45,46]. Very often, an exhaustive determination of all LM and TS is practically impossible and not strictly necessary, particularly when the number of degrees of freedom is very large. In these cases, a representative set of LM and TS is determined, on the basis of which the static and dynamic physical properties of the system are obtained. In this work the LM and TS are calculated by means of the following algorithm, which is based on a series of single-ended transition state searches adapted from Ref. [47]: (i) Start by choosing a local minimum from the database that has not yet been used for locating transition states. (ii) Perform an eigenvector-following search along a specific eigenvector of the Hessian $\hat{H}$ at this local minimum. Usually, one chooses eigenvectors corresponding to the smallest eigenvalues of $\hat{H}$, since the energy increase along these directions is smallest *a priori*. (iii) Once a transition state is found, the two adjacent local minima are identified by stepping off the transition state in the directions parallel and antiparallel to the single unstable mode. In this step, L-BFGS minimizations are performed [48]. (iv) If one of the two local minima corresponds to the initial local minimum, the other local minimum and the transition state are added to the database of stationary states. If not, the LM-TS-LM triplets are discarded, since we aim for a connected (ergodic) network of stationary states. (v) The algorithm proceeds by choosing a different eigenvector and repeating the steps (ii)–(iv). Once a specified number of eigenvectors have been tried (in the present case 15), the algorithm goes back to step (i) and a new LM is considered. The algorithm terminates after all local minima in the database have been used as initial states.

### 2.3. Kinetic Networks

The local minima and transition states of a given energy landscape form an *a priori* ergodic network that is known as the kinetic network (KN) of the system. It can be represented naturally by an undirected graph, in which the nodes represent the local minima and the edges symbolize the connecting pathways through the corresponding transition states. A number of parameters can be calculated in order to quantify and better understand the connectivity of a kinetic network and to compare the topology of the ELs of different physical systems.

One of the basic local network parameters is the degree $N_{c}\left(i\right)$ of each node *i*, which is defined as the number of links between node *i* and any other node of the network. The kinetic networks of different systems can vary widely in their size and structure. In the case of finite systems, finite simulation cells or when a natural correlation length is present, it is meaningful to introduce the local connectivity density

(2) $$\rho_{c}\left(i\right)=\frac{N_{c}\left(i\right)}{N_{LM}-1}$$

of node *i*, where $N_{LM}$ is the number of LM or nodes in the network. Nodes *i* with comparatively large values of $\rho_{c}\left(i\right)$ are often referred to as hubs: they play a central role in conveying the stochastic dynamics of complex systems.

Another useful network parameter is the distance $d_{ij}$ between two nodes *i* and *j* in a graph, which is defined as the smallest number of edges required to connect them. In other words, $d_{ij}$ gives the number of elementary transitions that are required to bring the system from metastable state *i* to metastable state *j*. The average path distance $\bar{d}$, obtained by averaging $d_{ij}$ over all pairs of nodes, gives thus a measure of the overall extension of the graph. In particular, if $\bar{d}$ is small, only a few elementary transitions are needed for the system to relax from any excited configuration towards the low-energy configurations or even to explore the complete configurational space. Conversely, as $\bar{d}$ increases, the dynamics become more complex and usually slows down, since a larger number of transitions are involved in most relaxation processes. The distance matrix $d_{ij}$ allows us to gain information on the location of the nodes within the kinetic network. In particular, the eccentricity $\varepsilon_{i}$ of a node *i*, defined as the largest $d_{ij}$ between node *i* and any other node *j*, measures the extent to which the node can be regarded as central or peripheral. Further interesting related properties are the radius *R* of the network, given by the minimum value of $\varepsilon_{i}$ among all nodes *i*, and the diameter *D*, which is the maximum $\varepsilon_{i}$. Note that neither *R* nor *D* are average properties of the network. In fact, they are prone to strong fluctuations among different comparable systems, since they are defined by the connectivity of one particular node.

Finally, it is often necessary to quantify the degree of clustering in a network. Indeed, knowing if the neighbors of a node are also neighbors of each other, provides insights on the short-time dynamics of the system. The clustering of a network is usually measured by the transitivity

(3) $$C=\frac{3\times \;\mathrm{number}\;\;\mathrm{of}\;\;\mathrm{triangles}}{\mathrm{number}\;\;\mathrm{of}\;\;\mathrm{triads}},$$

which represents the probability that in a triad of nodes, where *i* is connected to *j* and *j* is connected to *k*, also *i* and *k* are connected with each other [49].

For the purpose of future comparison, it is useful to direct our attention to two paradigmatic reference network models: periodic lattice structures and random graphs, which have strongly contrasting properties. In a random graph, the nodes are connected randomly with each other with a given average degree [50,51]. They have a relatively short average path distance $\bar{d}$ and a small transitivity *C*. In a lattice graph, on the other hand, each node has the same number of neighbors resulting in a large average path distance and a large transitivity or number of short loops. A particular combination of these properties leads to the notion of small-world network. According to Watts and Strogatz, a network can be regarded as a small world if it combines the short average path distance of random graphs with the large transitivity of lattice graphs [52]. Small-world networks are also characterized by the presence of hubs with large local connectivity densities. Many naturally occurring networks are small worlds, for instance, social and neural networks. In our context, it is interesting to elucidate if or under what circumstances, the kinetic networks of disordered NP ensembles exhibit small-world behavior [50,51].

### 2.4. Disconnectivity Graphs

The main limitation of kinetic networks as a tool for energy-landscape characterization is the lack of information on the energies of the connected local minima and the energy barriers separating them. Hence, they offer no clue about the time scales of the associated dynamics. Disconnectivity graphs (DGs), as proposed by Karplus et al. [53], provide this information and are therefore a much-used tool in the analysis of energy landscapes. Examples of the disconnectivity graphs of magnetic nanostructures are shown in Section 3. The physical meaning of these representations of the ELs can be clarified by describing how they are constructed [45,53].

The disconnectivity graph of a given connected network of LM and TS is created according to the following algorithm: For any given energy *E*, all energetically accessible local minima, i.e., the local minima having an energy lower than *E*, are grouped into disjoint sets denoted as superbasins. Two LM belong to the same superbasin, if there is a path, for example, the minimum energy path (MEP), which connects the two LM without exceeding the energy E. Usually one starts by choosing *E* close to the energy of the global minimum. In the absence of degeneracies, there is only one superbasin that contains the global minimum for such low values of *E*. If the ground state is *N*-fold degenerate, there are then *N* disjoint superbasins. As the energy *E* is gradually increased, more local minima become energetically accessible. Since in most cases these newly accessible LM are not all connected to the ground state without exceeding the increases *E*, one usually observes that the number of superbasins first increases. However, at some point, the superbasins start to merge with each other, since the separating energy barriers along the connecting MEP can be overcome. Eventually, for very high values of *E*, only one superbasin is left, which contains all local minima of the system, provided that the energy barriers are finite.

In practice, DGs are created by performing this algorithm at a discrete set of equidistant energies, which are indicated on the *y*-axis. At each energy *E*, a superbasin is represented by a node. Two nodes are connected with each other, if they share at least one local minimum. The horizontal position of the nodes is arbitrary. It is usually chosen in a way that nodes separated by lower energy barriers are closer to each other than those separated by larger energy barriers. In the end, the result is a tree-like graph, where the end point of each branch gives the energy of the corresponding local minimum and the merging of two branches indicates the energy barrier, which separates them (see, for instance, the DGs in Section 3).

### 2.5. Markovian Dynamics

At small temperatures, the transitions between different metastable states of the magnetic nanostructures can be regarded as rare events. In this case, the time evolution of a system, for instance, after magnetic saturation and external field removal or after a sudden temperature quenching, can be regarded as consisting of a series of independent elementary transitions, which are separated by long thermalization periods. The resulting Markovian dynamics can then be described by solving the master equation

(4) $$\frac{d\vec{P}}{dt}=Q\vec{P}$$

where the *i*-th component of $\vec{P}$ represents the occupation probability of the metastable state or basin *i*. The transition-rate matrix Q is given by

(5) $$Q_{ij}=k_{ij}-\delta_{ij}\sum_{l=1}^{\mathrm{LM}}k_{li}$$

where $k_{ij}$ stands for the transition rates for going from state *j* to state *i*. The solution of this system of linear differential equations can be expressed in terms of the eigenvalues $\lambda_{n}$ and the corresponding eigenvectors ${\hat{u}}_{n}$ of Q as

(6) $$\vec{P}\left(t\right)=\sum_{n=1}^{N_{\mathrm{LM}}}c_{n}\;e^{\lambda_{n}t}\;{\hat{u}}_{n},$$

where $c_{n}=\vec{P}\left(0\right)\cdot {\hat{u}}_{n}$ is the projection of the initial probability distribution $\vec{P}\left(0\right)$ on the eigenvector ${\hat{u}}_{n}$.

In this work, the transition rates $k_{ji}$ are calculated within the framework of harmonic transition-state theory (HTST) [54]:

(7) $$k_{ji}=a_{ji}\;\exp\left(-\frac{\Delta E_{ji}}{k_{B}T}\right),$$

where $a_{ji}$, usually denoted as attempt frequency, depends on the curvature of the energy landscape at the initial local minimum and at the separating transition state [54,55,56]. Typical values of $a_{ji}$ in magnetic nanostructures are in the range of $10^{8}$–$10^{11}\;\mathrm{Hz}$ [44,57]. In the absence of Goldstone modes, the temperature dependence of the expression solely resides in the Arrhenius exponential, which is defined by the separating energy barrier $\Delta E_{ji}$. Further details on the calculation of $k_{ij}$ may be found in Refs. [44,54,55,56].

## 3. Results

The goal of this section is to quantify the role of structural disorder on the energy landscapes of dipole-coupled magnetic nanoparticle ensembles, not only concerning the general trends, but also by giving particular emphasis to the fluctuations between different realizations having the same degree of disorder. For this purpose, four different representative realizations of two very different types of lattices are investigated in some detail: weakly-disordered square lattice (WDSL) ensembles, which are are obtained by starting from a perfectly periodic square lattice arrangement and applying small random displacements to the NP positions, and strongly disordered (SD) ensembles, in which the particles are randomly distributed within the unit cell without any overlap. The extended nanostructures are then modelled with finite unit cells having N=36 MNPs and periodic boundary conditions. The surface coverage is in all cases c=0.44, which corresponds to an average nearest-neighbor distance $r_{0}=4\;\mathrm{nm}$. Reasonable changes in the sample parameters are not expected to have a significant impact on our conclusions [19,20,21,44].

### 3.1. Weakly-Disordered Square Lattice Ensembles

#### 3.1.1. Ground-State Magnetic Order

**Figure 1:**
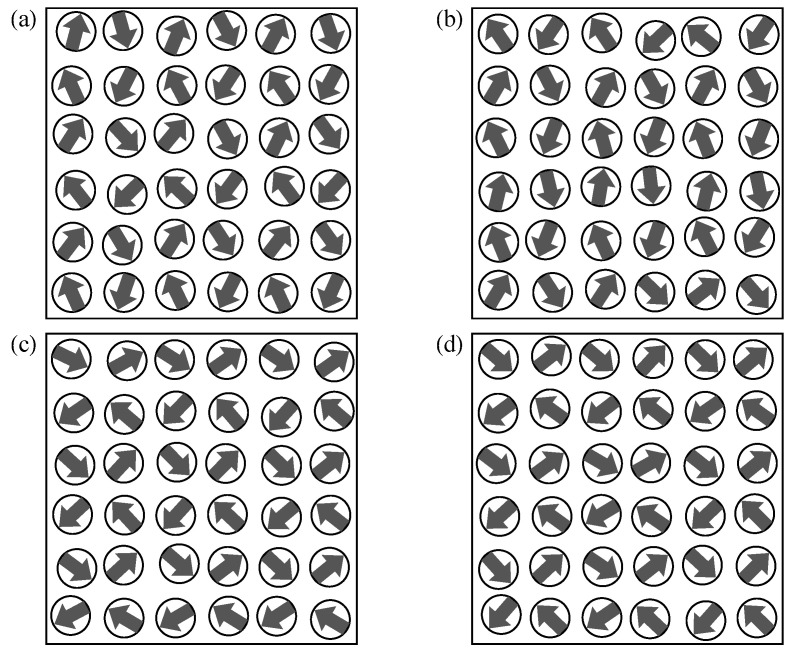
Ground-state magnetic configurations of four representative weakly-disordered square-lattice ensembles (**a**–**d**) having $\sigma_{r}=0.05\;r_{0}$. The positions of the MNPs within the unit cell are indicated by the disks and the directions of the magnetic moments by the arrows. Notice that all ground-state magnetic configurations correspond to a microvortex state with different angles $\alpha_{\mathrm{MV}}$ [see Equation (8)].

The ground-state magnetic configurations of a significant number of (more than 200) weakly-disordered square lattice ensembles with the same degree of structural disorder $\sigma_{r}=0.05\;r_{0}$ have been explored. In Figure 1, the NP positions within the unit cell and the ground-state magnetic configurations of representative realizations are shown. As a consequence of the strong out-of-plane anisotropy of the dipole interaction, the magnetic moments of the NPs always lie within the xy-plane as indicated by the arrows [15,44]. One observes that the ground-state magnetic configurations exhibit a long-range order, which is very close to the so-called microvortex (MV) state. Ignoring some minor random deviations due to disorder, the orientations of the magnetic moments ${\vec{m}}_{k}$ at each NP *k* are given by

(8) $$\begin{array}{c} m_{k}^{x}={\left(-1\right)}^{n_{y}}\;\cos\left(\alpha_{\mathrm{MV}}\right)\;\left|{\vec{m}}_{k}\right|\\ m_{k}^{y}={\left(-1\right)}^{n_{x}}\;\sin\left(\alpha_{\mathrm{MV}}\right)\;\left|{\vec{m}}_{k}\right|, \end{array}$$

where $\alpha_{MV}\in \left[0,\pi \right]$ is the microvortex angle, and $n_{x}$ and $n_{y}$ indicate the position of nanoparticle *k* along the *x* and *y* directions of the underlying square lattice [15]. For instance, $\alpha_{\mathrm{MV}}=0$ represents a striped antiferromagnetic state whereas $\alpha_{\mathrm{MV}}=\pi /4$ corresponds to a perfect vortex state.

In the perfectly periodic case without any disorder, the ground state is continuously degenerate with respect to $\alpha_{\mathrm{MV}}$, which can be shown to be a consequence of the $C_{4}$ rotational symmetry of the square lattice [14,58,59]. This continuous degeneracy is broken by the slightest degree of structural disorder. As a result, one or a few specific values of $\alpha_{\mathrm{MV}}$ are stabilized with respect to all others, as can be seen in the NP ensembles illustrated in Figure 1. In these particular cases, $\alpha_{\mathrm{MV}}$ adopts values between 0.55 in (d) and 1.09 in (b). This long-range-order stabilization is generally known as order-by-disorder effect [14,60,61,62]. The only remaining symmetry is then time-reversal, which implies that the ground state (and any other magnetic configuration) has the same energy as the one obtained by reversing all spin directions.

From the previous considerations, one might be tempted to conclude that the ground-state values of $\alpha_{\mathrm{MV}}$ bear no correlation between different NP ensembles having the same degree of disorder and that it adopts all values in [0,π] with equal probability. However, the theoretical work by Prakash et al. has shown that structural disorder systematically favors MV angles close to $\alpha_{\mathrm{MV}}=\pi /4=0.785$ [14,59]. This remarkable result is a consequence of the fact that, although the energy *E* in the periodic square lattice is independent of $\alpha_{\mathrm{MV}}$, the curvature of the energy landscape (or in more physical terms the spin-wave density) very much depends on it. Therefore, MV states having different $\alpha_{\mathrm{MV}}$ behave differently under the influence of structural disorder. In our case the calculated average of $\alpha_{\mathrm{MV}}$ of 100 realizations is ${\bar{\alpha }}_{\mathrm{MV}}=0.77\pm 0.35$, which is close to the theoretically expected value even if the standard deviation is still significant. These deviations are possible a consequence of the finite size of the considered unit cells of the investigated ensembles, which preclude full self-averaging. The specific examples shown in Figure 1 allow us to asses the measure of the fluctuations of $\alpha_{\mathrm{MV}}$ for different, hardly discernible but visually equivalent ensembles.

#### 3.1.2. Kinetic Networks

**Figure 2:**
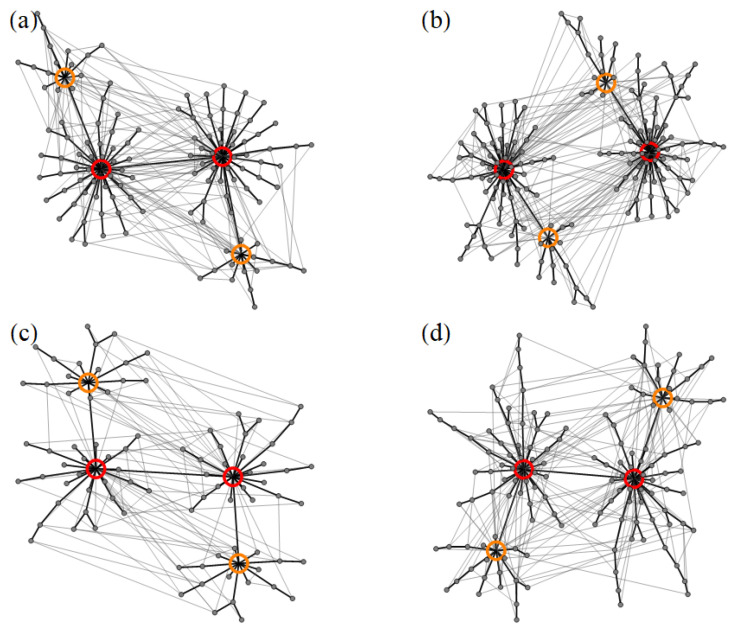
Kinetic networks of the local minima and transition states of the weakly-disordered square ensembles (**a**–**d**) illustrated in Figure 1. The two-fold degenerate ground states are indicated by red circles. Additional hubs are indicated by orange circles. The highlighted black segments correspond to the transition which leads from a given minimum to a minimum having a lower or equal energy by involving the smallest energy barrier. The number of local minima and transition states of each NP ensemble can be found in Table 1.

Previous studies have shown that the energy landscapes of disordered ensembles of dipole-coupled magnetic nanoparticles contain a large number of local minima, which are connected through diverse transition states [19,20,21,44]. Each LM-TS-LM triplet represents an single elementary transition or relaxation process which, together, define the dynamics of the system. The connectivity among the LM is displayed by the kinetic networks of Figure 2. They correspond to the NP ensembles illustrated in Figure 1. As usual, the nodes represent the LM and the connecting TS are indicated by grey edges. The black edges highlight the dynamically most dominant relaxation process for each LM, which are those involving the lowest energy barrier while leading to a LM with lower or equal energy [44]. One observes that the ground states (red circles) are always at the center of the kinetic networks. They are directly connected to an important fraction of the LM, as measured by their large connectivity density $\rho_{c}$, which ranges from $\rho_{c}=0.38$ in (d) to $\rho_{c}=0.45$ in (a). Hence, the ground states are clear hubs of the kinetic networks of weakly-disordered NP ensembles (see Figure 2). The large values of $\rho_{c}$ can be attributed to the huge basins of attractions of the ground states. This are a consequence of lifting the continuous degeneracy of the MV state in the periodic lattice by means of structural disorder [44,63]. Besides the ground state, one finds in almost all cases at least two degenerate additional LM that are hubs (orange circles) which also stem from the MV manifold, but which have a slightly higher energy. Most importantly, the KNs show that the dynamically dominant transitions are all channeled towards the hubs, which thus act as veritable funnels of the networks. One concludes that the ELs of weakly-disordered square-lattice ensembles share their main characteristics with other good structure-seeking systems such as good-folding proteins and magic-number clusters [44,64,65,66].

One of the main difference between the ELs of different WDSL ensembles lies in the number of local minima $N_{\mathrm{LM}}$. In Table 1 results for $N_{\mathrm{LM}}$ are given for the nanostructures illustrated in Figure 1. Even though $N_{\mathrm{LM}}$ ranges between $N_{\mathrm{LM}}=80$ in (c) to $N_{\mathrm{LM}}=148$ in (b), the network topology is not significantly affected (see Figure 2). Moreover, the main network parameters given in Table 2, namely, the average path distance *d*, radius *R*, diameter *D*, transitivity *C* and ground-state connectivity density $\rho_{c}$, are all very similar or the same. This is a consequence of the fact that most of the differences among the various realizations of disorder occur at the periphery of the kinetic networks rather than at the center. Therefore, the general properties of the networks are mostly unaffected. The equilibrium and dynamical properties of these NP ensembles should be very similar, even though $N_{\mathrm{LM}}$ varies to some extent between different realizations. Concerning the values of *d*, *R*, and *D*, it is important to note that they are relatively small, which is consistent with the afore discussed good-structure-seeker behavior and with the large ground-state connectivity density (see Table 2 and Ref. [44]).

Finally, it is always of great interest to investigate, whether a physically meaningful network exhibits small-world properties by comparing its average path distance *d* and transitivity *C* with those of random graphs having the same number of nodes and edges. A previous study has already shown that kinetic networks of weakly-disordered square lattice ensembles do exhibit small-world behavior [44]. Yet, the question remains to quantify to what extent the network parameters defining small-world behavior are affected by the details in the nanostructure realizations. The results shown in Table 2 clearly indicate that the network behavior is remarkably robust. Not only is the average path length *d* significantly smaller than in the corresponding random graphs, but also the transitivity *C* is always significantly larger. This, together with the presence of large hubs, convincingly shows that the kinetic networks of WDSL ensembles are small worlds. Similar conclusions are drawn by considering the averages over a large number of realizations.

#### 3.1.3. Disconnectivity Graphs

**Figure 3:**
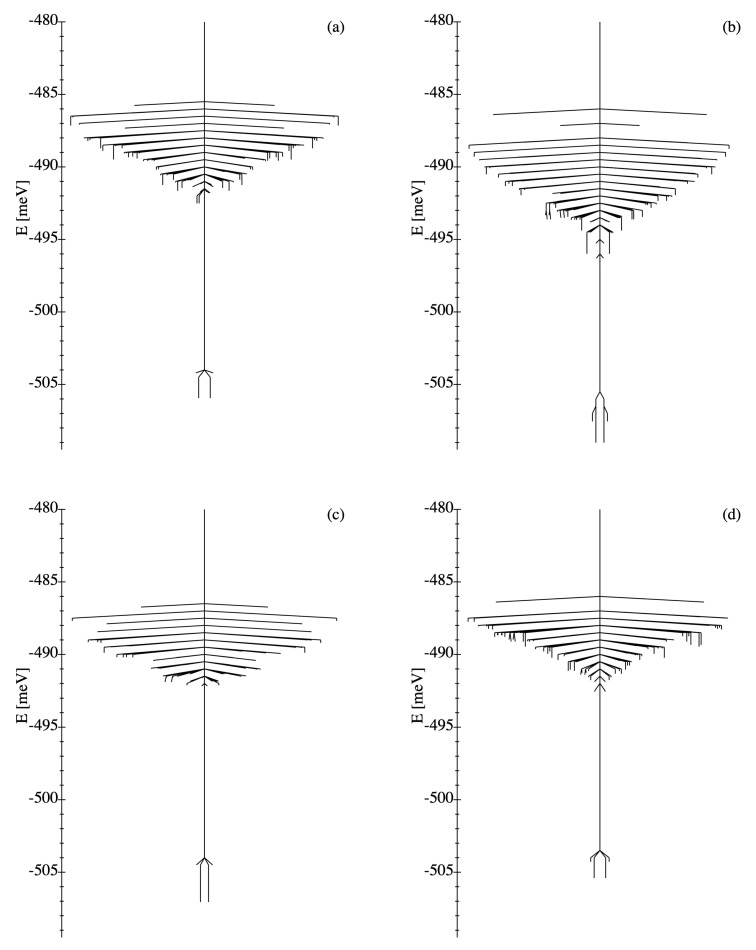
Disconnectivity graphs of the energy landscapes of the four weakly-disordered square lattice ensembles (**a**–**d**) illustrated in Figure 1. The two-fold degeneracy of all local minima is a known consequence of time-reversal symmetry.

A complementary perspective on the energy landscapes of WDSL ensembles is provided by the disconnectivity graphs shown in Figure 3. Here, the focus is no longer on the connectivity among the local minima, but rather on their energies and in particular on the energy barriers separating them, which are most important from a dynamical perspective. First of all, one observes, as in the kinetic networks discussed in Section 3.1.2, that the disconnectivity graphs of the different NP ensembles are qualitatively very similar. In all cases, one finds a clearly identifiable ground state together with a very small number of excited magnetic configurations, which have close-by energies and which correspond to MV states with different microvortex angles $\alpha_{\mathrm{MV}}$ (see Section 3.1.2). All the other excited states are located at much larger energies. Furthermore, the energy profiles in the disconnectivity graphs are extremely asymmetric, since the energy barriers leading towards the two ground states are very small, while the energy barriers in the opposing direction are much larger. Consequently, a very fast relaxation dynamics is expected in agreement with the previously discussed kinetic networks. The results confirm the conclusion that weakly-disordered square ensembles of dipole-coupled MNPs are good structure-seekers regardless of the specific realization of disorder [44].

Concerning the differences between the individual realizations, the DGs of Figure 3 show that they occur mostly in the high-energy part of the spectrum. Therefore, despite the fact that the number of local minima $N_{\mathrm{LM}}$ varies between the different realizations, one does not expect any significant differences in the equilibrium and dynamical properties at low temperatures. Quantitatively, the energy barrier between the two ground states varies from ΔE=1.64meV in (d) to ΔE=3.39meV in (b). While this would not affect the physical properties of the nanostructures, it conditions some aspects of the relaxation dynamics, for example, if the system is prepared in an asymmetric initial state.

#### 3.1.4. Relaxation Dynamics after Quenching

Numerical simulations of the magnetic relaxation of weakly-disordered square-lattice ensembles of magnetic NPs have been performed in order to demonstrate the correlation between the characteristics of the ELs discussed in the previous sections and the actual magnetic response of the corresponding magnetic nanostructures. The considered experimental situation or simulation protocol consists in an initial thermalization of the nanostructures at a relatively high temperature $T^{*}=200\;K$, which is larger than most of the energy barriers in these systems ($k_{B}T^{*}=17.23\;\mathrm{meV}$, see Figure 3). At time t=0, once the system has reached equilibrium at $T^{*}$, it is rapidly quenched to a much lower temperature *T*, which is kept constant throughout the simulation. The relaxation dynamics is subsequently recorded until the new equilibrium state is reached. This simulation protocol, which we refer to as relaxation after quenching (RAQ), corresponds to a symmetric (isotropic) initial probability distribution $\vec{P}\left(0\right)$, which is completely defined by the canonical equilibrium state at $T^{*}$.

The time-dependence of the long-range magnetic order in the nanostructures is quantified by the microvortex order parameter

(9) $$\eta_{\mathrm{MV}}={\tilde{M}}_{x}^{2}+{\tilde{M}}_{y}^{2},$$

where

(10) $${\tilde{M}}_{x}=\frac{1}{Nm}\sum_{n_{x}n_{y}}{\left(-1\right)}^{n_{y}}m_{n_{x}n_{y}}^{x}\;\mathrm{and}\;{\tilde{M}}_{y}=\frac{1}{Nm}\sum_{n_{x}n_{y}}{\left(-1\right)}^{n_{x}}m_{n_{x}n_{y}}^{y}$$

are the components of the staggered magnetization adapted to the MV state and $m_{n_{x}n_{y}}^{x}$ ($m_{n_{x}n_{y}}^{y}$) is the *x* (*y*) component of the magnetic moment of the NP located at the $n_{x}$-th row and $n_{y}$-th column (see Section 3.1.1 and Ref. [15]). This is the most interesting order parameter for square-lattice arrangements as the ground-state configuration is close to the perfect microvortex state which has $\eta_{\mathrm{MV}}=1$ (see Section 3.1.1). A further important property characterizing the approach to equilibrium is the configurational entropy, which is defined by

(11) $$S=-k_{B}\sum_{i=1}^{N_{\mathrm{LM}}}P_{i}\;\ln\;P_{i},$$

where $P_{i}$ is the occupation probability of the metastable state *i*. Equation (11) gives a measure of the diversity of the probability distribution of the system throughout the energy landscape. However, notice that the vibrational-like entropy associated with the fluctuations of the NP magnetic moments within each basin of attraction is disregarded at this stage.

**Figure 4:**
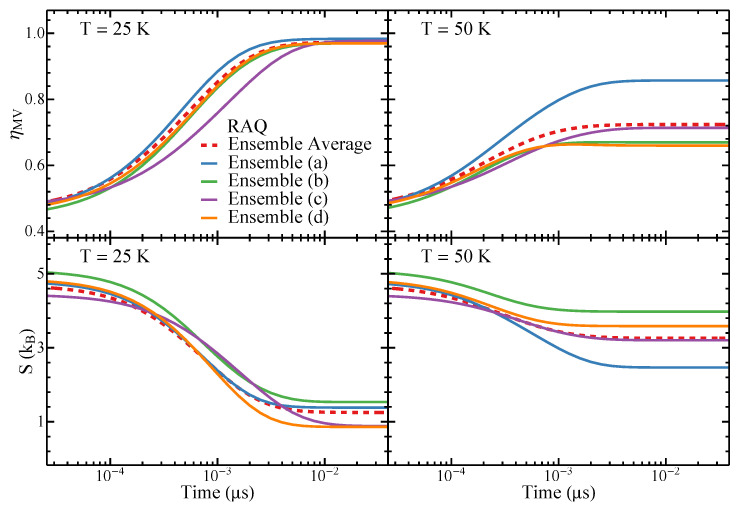
Time dependence of the microvortex (MV) order parameter $\eta_{\mathrm{MV}}$ and the configurational entropy *S* of the WDSL ensembles illustrated in Figure 1 (solid curves) following a relaxation after quenching (RAQ). The corresponding averages over 100 different realizations of the nanostructures are given by the dashed curves. The simulation temperatures *T* are indicated.

In Figure 4, the time-dependence of $\eta_{\mathrm{MV}}$ and *S* in the WDSL ensembles illustrated in Figure 1 are displayed for T=25K and T=50K. For the sake of comparison, the average over 100 different realizations is also shown (dashed curves). One observes that for all ensembles $\eta_{\mathrm{MV}}$ increases monotonously with time *t* whereas *S* decreases. This behavior is a consequence of the fact that the low-energy states, which have a strong MV order, are increasingly occupied at the expense of high-energy states, which are magnetically disordered to a large extent. At T=25K, only the lower-energy states are significantly occupied in the final equilibrium state. This results in values of $\eta_{\mathrm{MV}}$ which are close to unity and in relatively small values of *S*. In contrast, at T=50K the excited states remain appreciably occupied even in the equilibrium state, which leads to clearly smaller values of $\eta_{\mathrm{MV}}$ and larger values of *S*.

Furthermore, one observes that the time evolution is close to exponential in all the investigated ensembles. There are no signs of complicated non-equilibrium effects such as trapping and slowing-down. This is in agreement with our analysis of the kinetic networks and disconnectivity graphs of the energy landscapes as well as with previous studies of WDSL ensembles [44]. Hence, the simulations of the relaxation dynamics confirm that WDSL ensembles are good structure-seeking systems with unhindered fast relaxation dynamics.

The differences between individual ensembles are relatively small. In particular, the relaxation time scales are nearly the same in all ensembles. Thus, the overall time evolutions are close to the one obtained by averaging over a large number of different nanostructure realizations. The same holds for the equilibrium values of $\eta_{\mathrm{MV}}$ and *S*, showing that the considered ensembles, their energy landscapes and their dynamics are indeed representative of WDSL ensembles in general. Only ensemble (a) shows slightly stronger deviations for $\eta_{\mathrm{MV}}$ and *S* at T=50K. This can be traced back to the energy differences between the low-energy states and the higher-energy states, which are slightly larger in this ensemble than in the others. This results in more stable low-energy states and thus in larger values of $\eta_{\mathrm{MV}}$ and smaller values of *S* at increasing temperatures.

### 3.2. Strongly-Disordered Ensembles

#### 3.2.1. Ground-State Magnetic Configurations

**Figure 5:**
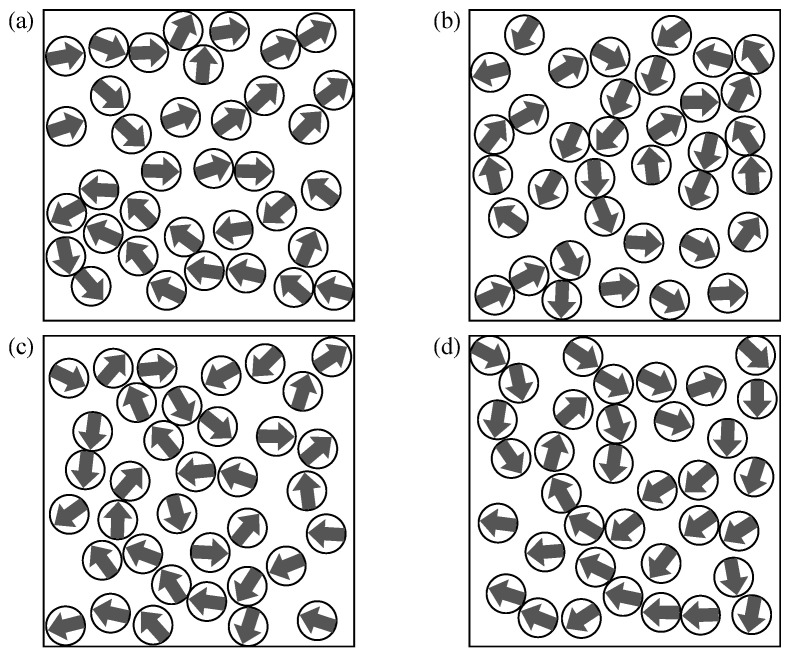
Ground-state magnetic configurations of representative strongly-disordered ensembles (**a**–**d**) of MNPs. The positions of the particles within the unit cell are indicated by the disks and the directions of the magnetic moments by the arrows. Notice that the magnetic configurations are dominated by short-range correlations between the local moments.

The magnetic properties of 100 different strongly-disordered dipole-coupled nanoparticle ensembles have been investigated. The NP positions and the ground-state magnetic configurations of four representative realizations are shown in Figure 5. In contrast to WDSL ensembles, no clear signs of long-range order can be recognized. Instead, the ground-state magnetic configurations are primarily dominated by short-range head-to-tail orientations of neighboring magnetic moments, which are energetically the most favorable. In addition, depending on the local environments of the NPs, other kinds of magnetic arrangements are also found, for example, vortices, branchings, as well as some ferromagnetic domains of variable size (see Figure 5).

A further important characteristic of the ground state of SD ensembles is that the magnetic order changes widely among the various realizations of disorder. No common long-range order can be recognized by comparing the different SD ensembles. This contrasts with the MV state found in WDSL ensembles or the ferromagnetic order in weakly-disordered triangular lattices [16,44,67]. Nevertheless, on a short length scale, qualitatively similar magnetic structures can be identified (e.g., head-to-tail, vortex-like, etc.) which are arranged in different ways depending on the NP locations. It seems therefore not possible to describe or classify these configurations by means of a single global order parameter, which would reflect the hierarchy among the different metastable states in a meaningful way. For instance, even though the total magnetization *M* is expected to be relatively small in the low energy states in order to minimize magnetic stray fields, we find that it fluctuates very strongly from M=0.02m in the ground state of ensemble (d) to M=0.15m in (c), where *m* stands for the NP moment.

#### 3.2.2. Kinetic Networks

**Figure 6:**
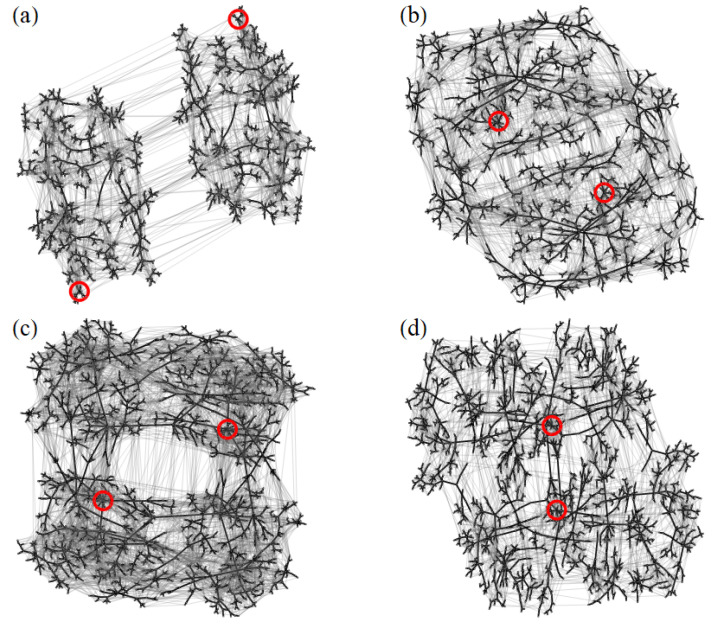
Kinetic networks of the local minima and transition states of the strongly-disordered ensembles (**a**–**d**) illustrated in Figure 5. The two-fold degenerate ground states are indicated by red circles. The highlighted black segments indicate the transitions, which connect each minimum with a minimum having a lower or equal energy by involving the smallest energy barrier. The number of local minima and transition states of each NP ensemble may be found Table 3.

A clearer insight into the morphology of the energy landscapes of SD ensembles can be obtained by analyzing the kinetic networks shown in Figure 6. The corresponding numbers of local minima $N_{\mathrm{LM}}$ and transition states $N_{\mathrm{TS}}$ can be found in Table 3. A number of contrasting differences with respect to the kinetic networks of WDSL ensembles deserve to be stressed. Not only $N_{\mathrm{LM}}$ and $N_{\mathrm{TS}}$ are much larger in SD ensembles, but also the topology of the kinetic networks itself is far more complex, even if we restrict ourselves to the dynamically dominant transitions which are highlighted in black. Furthermore, the ground states (red circles) are no longer hubs of the kinetic networks. Their connectivity density is only $\rho_{c}=0.01$, which is much smaller than in the WDSL case. In fact, the kinetic networks of SD ensembles have no hubs at all. Instead, these networks are diverting and tend to decompose into smaller subnetworks. Consequently, a highly complex relaxation dynamics is expected, which involves many intermediate metastable states and which is not funnelled towards a clear set of low-lying magnetic configurations. These findings differ strikingly from the trends observed in weakly-disordered arrangements (cf. Figure 2 and Figure 6). The specific behavior of SD ensembles is also clearly demonstrated by the different topological network parameters. For instance, the average path distances in the illustrated ensembles vary between d=6.0 in (d) and d=6.6 in (a), which are much larger than in the WDSL ensembles. The same holds for the radius *R* and diameter *D*. However, the transitivities are comparable (cf. Table 2 and Table 4).

The strong increase of $N_{\mathrm{LM}}$ in SD ensembles can be attributed to the shorter correlation lengths between the NP moments [21,44]. In fact, in WDSL ensembles the correlation length between the NP moments is extremely large, since almost all elementary transitions involve reorientations of nearly all NP moments at the same time. This results in a comparatively small number of metastable states with extended basins of attraction. In contrast, the correlation length in SD ensembles is much shorter, since it most often involves only one NP and its immediate environment. This allows for a multitude of elementary transition in which only a small number of NP moments change direction [21]. The basins of attraction of the LM in SD ensembles are accordingly much smaller. Moreover, one observes that increasing the size of the unit cells in WDSL ensembles only leads to a modest increase of $N_{\mathrm{LM}}$, whereas $N_{\mathrm{LM}}$ in SD ensembles increases almost exponentially with the unit-cell size. This is a consequence of the fact that different local arrangements and domains within the magnetic configurations of SD ensembles are nearly statistically independent [68].

Concerning the differences between the kinetic networks of different SD ensembles, Figure 6 shows that the overall network topology is quite similar qualitatively. This is also reflected by the network parameters *d*, *R*, *D*, and *C* reported in Table 4, which are all very much alike. An important difference between the kinetic networks stems, however, from time-reversal symmetry and from the way in which symmetry-equivalent magnetic configurations are related. The two-fold degeneracy of each state leads to a splitting of the networks in two kind of hemispaces, which can clearly be identified in each of the networks shown in Figure 6. The extent of the splittings depends on the particular ensembles. For instance, in ensemble (a) the splitting is very strong and the two time-inversion related hemispaces are nearly disconnected from each other. The scarceness of transitions results in a slowing down of the thermalization between them. In contrast, in the ensemble (b) the splitting between hemispaces is much weaker. The reason behind such differences lies probably in the presence of particularly stable magnetic configurations, which do not change significantly at a local level, i.e., among directly connected metastable states. Reversing all the local moments in one of them, in oder to reach its time-inverted image, requires multiple elementary transitions, which renders the connectivity between hemispaces particularly difficult. Depending on the stability of these magnetic configurations, the splitting is stronger in some ensembles and weaker in others.

Finally, before closing this section, it is worth analyzing the topology of the kinetic networks SD ensembles with respect to small-world behavior. The results in Table 4 show that the average path distance *d* and transitivity *C* are always larger in the kinetic networks of SD ensembles than in the random graphs having the same number of nodes and edges. This means that the small-world criterion of Ref. [52] is not satisfied. Moreover, the complete lack of hubs is a further clear indication that the kinetic networks shown in Figure 6 do not exhibit small-world properties, thus confirming the conclusions of previous studies [44]. A similar behavior has also been observed in other kinds of systems, such as clusters and glasses, which also show slow and complex relaxation dynamics [63].

#### 3.2.3. Disconnectivity Graphs

**Figure 7:**
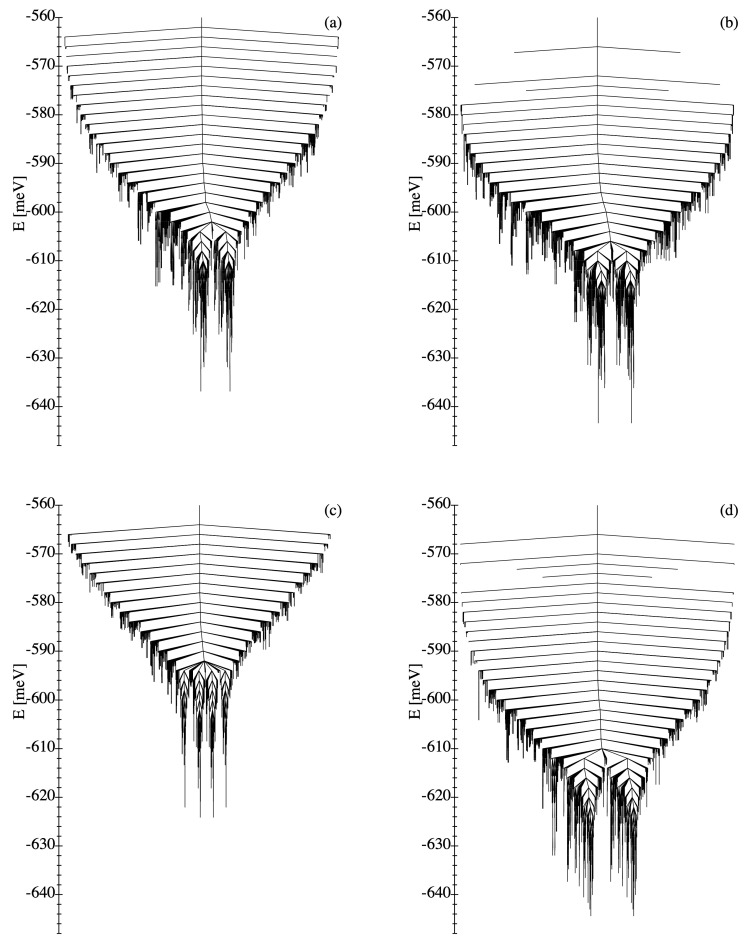
Disconnectivity graphs of energy landscapes of the strongly-disordered ensembles (**a**–**d**) illustrated in Figure 5. The two-fold degeneracy of the LM is a consequence of time-reversal symmetry.

As already observed in Section 3.1.3, the disconnectivity graphs provide a most useful complementary perspective to the ELs of MNP ensembles. In Figure 7 the DGs of the SD ensembles illustrated in Figure 5 are shown.

First of all, one is amazed by the high level of complexity that they reflect, which obviously surpasses that of WDSL ensembles. In addition to the much larger number of local minima at all energy levels, one also finds a particularly important number of low-lying LM whose energies are all very similar.

While the energy barriers surrounding high-energy LM are small, as in the weakly-disordered case, the barriers separating the low-energy LM are relatively large, often much larger than the energy differences between them. Hence, the relaxation dynamics from high-energy LM towards low-energy LM is expected to be relatively fast, whereas the thermalization among low-energy LM should be much slower and complex. Consequently, the magnetization dynamics of SD ensembles is not only significantly slower than in WDSL ensembles, but in addition it involves multiple time scales which tend to increase as the system evolves in time (e.g., as in a stretched exponential behavior) [44]. In fact, the DGs shown in Figure 7 closely resemble those found in structural glasses, which indicates that the dynamic behavior should be similarly intricate [44,69,70,71,72].

As already observed for the KNs, the differences between the DGs of various SD ensembles are significantly stronger than in the case of WDSL ensembles. In SD ensembles, the changes in the DGs occur not only in the high-energy parts of the spectrum, but also at the low energies, as can be clearly seen in Figure 7. Hence, one expects an important quantitative dispersion in the static and dynamic behavior among different realizations of disorder in the unit cell. However, since the general trends are very similar, no significant qualitative differences are expected.

#### 3.2.4. Relaxation Dynamics after Saturation

As in the case of WDSL ensembles, the link between the energy landscapes and relaxation dynamics of strongly-disordered nanostructures deserves to be established. In the case of SD ensembles, a particularly interesting experimental or simulation situation is the isothermal relaxation after saturation (RAS). Starting from a fully polarized configuration in an arbitrary direction, the magnetic field is turned off at time t=0. Consequently, the system falls into the closest LM *j* of the field-free energy landscape. In general, the magnetic configuration of this state is not fully polarized as it involves the barrierless part of the relaxation starting from the initial fully polarized state. This preparation protocol results in a quite asymmetric initial probability distribution $P_{i}\left(0\right)=\delta_{ij}$. Thus, the initial occupation probability is zero, except for one state *j*, which is defined by the saturating magnetic field direction, namely, the one whose basin of attraction includes the given saturated magnetic configuration. The long-range magnetic order in the SD ensembles is quantified best by the ferromagnetic (FM) order parameter $\eta_{\mathrm{FM}}$ which is simply given by

(12) $$\eta_{\mathrm{FM}}=M_{x}^{2}+M_{y}^{2}$$

where

(13) $$\vec{M}=\frac{1}{Nm}\sum_{k}{\vec{m}}_{k}$$

is the average magnetization of the system measured in units of the NP moment *m*.

**Figure 8:**
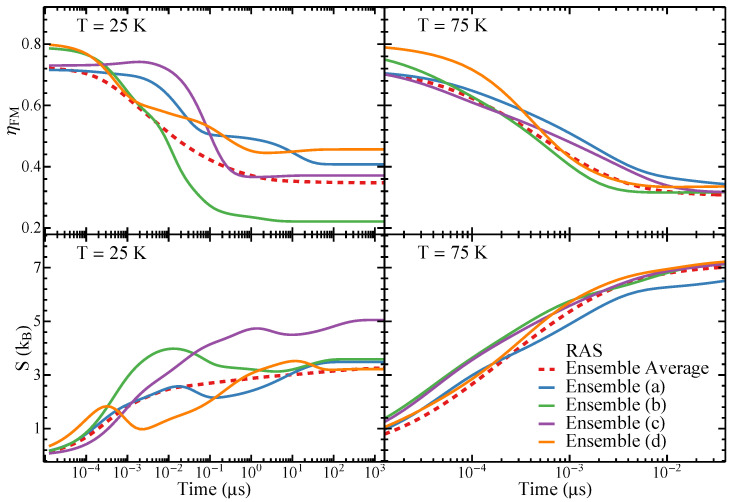
The time dependence of the ferromagnetic (FM) order parameter and of the configurational entropy *S* of the SD ensembles illustrated in Figure 5 (solid curves) following an isothermal relaxation after saturation (RAS) are shown. Moreover, the average of $\eta_{\mathrm{FM}}$ and *S* over 100 different realizations of the nanostructures are given by the dashed curves. The simulation temperatures are indicated.

Figure 8 shows the time dependence of the FM order parameter $\eta_{\mathrm{FM}}$ and of the configurational entropy *S* in the SD ensembles illustrated in Figure 5, together with the corresponding averages over 100 different realizations for the temperatures T=25K and T=75K. One observes that $\eta_{\mathrm{FM}}$ decreases with increasing *t* starting from a strongly polarized state where $\eta_{\mathrm{FM}}≃0.8$ is large. As the systems evolve in time out of strongly ferromagnetic states towards the low-energy configurations, $\eta_{\mathrm{FM}}$ decreases, since the magnetization in the low-lying states is usually small due to their tendency to magnetic flux closure (see Figure 5, where the ground-state magnetic configurations are illustrated). At the same time, the configurational entropy, which is equal to zero in the initial well-defined state, increases as other metastable states are increasingly populated.

It is important to note that the relaxation towards equilibrium of the SD ensembles is profoundly different from the behavior of WDSL ensembles (cf. Figure 4 and Figure 8). First, it takes place on a completely different time scale, which is orders of magnitude larger than in WDSL ensembles. Second, the time evolution at the lower temperature T=25K of the individual ensembles is not monotonous. Instead, remarkable non-equilibrium phenomena are found including in particular trapping, as indicated by the plateaus in $\eta_{\mathrm{FM}}$ and by some minima in *S* as a function of *t*. This is a clear indication that the equilibration within smaller regions of configurational space takes place on completely different time scales than the equilibration of large macroscopic systems, which is in agreement with the results shown in Section 3.2.1, Section 3.2.2 and Section 3.2.3 and in previous studies [44]. Finally, as the temperature is increased, for instance, to T=75K, the trapping effects and the differences between different realizations of disorder are strongly softened, since the details in the local NP arrangements and magnetic configurations become less relevant as the energy barriers responsible for trapping can be more easily overcome. Notice, in particular, that the relaxation time scales of different ensembles tend to be similar at T=75K, and yet they remain remarkable large in comparison with the weakly-disordered ensembles. This reflects the far more intricate nature of their ELs, as shown in Figure 6 and Figure 7.

As already mentioned in the discussion of the kinetic networks and disconnectivity graphs, the differences observed in the behavior of the various realizations of disorder are much stronger in SD ensembles than in WDSL ensembles. Indeed, trapping involves different magnetic states, it occurs at different times along the dynamics, and it lasts for different periods of time. Still, the main qualitative features and relaxation time scales remain relatively similar (see Figure 8). Thus, the average of the time dependence over different NP arrangements, which can be regarded as representative of extended NP ensembles, converges rather rapidly provided that the temperature is not too low. The calculated time dependences of $\eta_{\mathrm{FM}}$ and *S* for different strongly-disordered arrangements of the NPs give us a unique insight on the way self-averaging in extended nanostructures most probably takes place. In fact, in agreement with previous studies showing that the elementary relaxation processes in dipole-coupled NP ensembles become increasingly localized as disorder increases, we observe that different local arrangements of the NPs follow different sometimes even non-monotonous pathways towards equilibrium [21]. Thus, the relaxation of the different NP arrangements on small unit cells are expected to be representative of subsystems of larger macroscopic ensembles. Only the average of a large number of such local situations yields a monotonous convergence to equilibrium.

## 4. Conclusions

The energy landscapes of disordered two-dimensional ensembles of dipole-coupled magnetic nanoparticles have been investigated. The ergodic networks of local minima and connecting first-order saddle points have been determined for an important number of weakly-disordered square-lattice ensembles and strongly-disordered ensembles. The analysis shows that structural disorder is responsible for a remarkable transformation in the collective behavior of these nanostructures. Weakly-disordered square-lattice ensembles are good structure-seekers with a clearly identifiable time-inversion degenerate ground state and very few low-lying states, all of which have large basins of attraction to which the vast majority of higher-energy metastable configurations are directly or almost directly connected. It can therefore be anticipated that the system responds unhindered and relatively fast to changes of external parameters. In contrast, strongly-disordered ensembles have very rough and complex energy landscapes with a much larger number of local minima and a multitude of low-lying metastable magnetic configurations whose energy differences are much smaller than the energy barriers separating them. These energy landscapes resemble those found in glasses. They are characteristic of systems showing slow complex dynamical responses, which are prone to trapping and are likely to involve multiple relaxation time scales.

Once the energy landscapes have been characterized, we determined the Markovian dynamics of WDSL ensembles following an abrupt temperature quenching as well as the dynamics of SD ensembles following magnetic saturation. From a qualitative perspective, the results confirm the conclusions drawn from the calculated kinetic networks and disconnectivity graphs in all respects. Moreover, the possibility of contrasting the quantitative results for multiple realizations of disorder have allowed us to unambiguously demonstrate the profound correlations between the KNs and DGs of the energy landscapes on the one hand and the time evolution of the magnetic order η and configurational entropy *S* on the other hand. The system specific properties have been contrasted with the corresponding averages over a large number of nanoparticle arrangements. In particular in the case of SD ensembles, remarkable non-monotonous time-dependencies of η and *S* have been observed which provide new insights on the trapping effects occurring at different length scales.

The primary focus of this paper has been on the dipole–dipole interactions between the NPs and on the cooperative many-body effects resulting from the interplay between these interactions and structural disorder. In view of more comprehensive comparisons with experiment, it would be worthwhile to extend this work by taking other magnetic effects into account. This includes local contributions, such as the magnetocrystalline and shape anisotropies of individual magnetic nanoparticles and their dispersion, as well as global contributions, such as the coupling to external magnetic fields. The former, which are always present to some extent in experiment, are expected to reduce the spatial extent of the correlation between local moments of different NPs. The latter should modify the EL more profoundly by changing the number of local minima and inducing multiple catastrophes. Further interesting research directions to be pursued in the present framework concern exploring other types of interactions, for example, quadrupolar couplings between coated NPs, RKKY interactions mediated by a metallic support, and direct exchange couplings at the interfaces between NPs in contact.

## Figures and Tables

**Table 1:** Ground-state microvortex angle $\alpha_{\mathrm{MV}}$, number of local minima $N_{\mathrm{LM}}$ and number of transition states $N_{\mathrm{TS}}$ of the WDSL ensembles illustrated in Figure 1. The corresponding averages μ and standard deviations σ are obtained from 100 different realizations of disorder.

	(a)	(b)	(c)	(d)	μ±σ
$\alpha_{\mathrm{MV}}$	1.04	1.09	0.68	0.55	0.77±0.37
$N_{\mathrm{LM}}$	120	148	80	132	121±42
$N_{\mathrm{TS}}$	392	474	254	426	433±144

**Table 2:** Topological parameters of the kinetic networks of WDSL ensembles illustrated in Figure 2: average path distance *d*, radius *R*, diameter *D*, transitivity *C* and ground-state connectivity density $\rho_{c}$. The corresponding averages μ and standard deviations σ are obtained from 100 different realizations of disorder. For the sake of comparison, the  values in brackets indicate the results for random graphs having the same number of nodes and edges.

	(a)	(b)	(c)	(d)	μ±σ
*d*	2.51 (3.27)	2.64 (3.43)	2.63 (3.27)	2.67 (3.29)	2.47±0.20
*R*	3.00 (4.06)	3.00 (4.38)	3.00 (4.12)	3.00 (4.04)	2.64±0.54
*D*	5.00 (6.62)	5.00 (7.14)	5.00 (7.00)	5.00 (6.54)	4.60±0.77
*C*	0.08 (0.04)	0.08 (0.03)	0.11 (0.05)	0.09 (0.04)	0.10±0.03
$\rho_{c}$	0.45 (0.09)	0.43 (0.09)	0.40 (0.08)	0.36 (0.09)	0.48±0.11

**Table 3:** Total number of local minima $N_{\mathrm{LM}}$ and of transition states $N_{\mathrm{TS}}$ of the strongly-disordered NP ensembles illustrated in Figure 5. The corresponding averages μ and standard deviations σ are obtained for 100 different realizations of disorder.

	(a)	(b)	(c)	(d)	μ±σ
$N_{\mathrm{LM}}$	2276	2162	2596	2532	2628±1043
$N_{\mathrm{TS}}$	8876	7982	11,100	9700	10,389 ± 4468

**Table 4:** Topological parameters of the kinetic networks of SD ensembles illustrated in Figure 6: average path distance *d*, radius *R*, diameter *D*, transitivity *C* and ground-state connectivity density $\rho_{c}$. The corresponding averages μ and standard deviations σ are obtained for 100 different realizations of disorder. Results for random graphs having the same number of nodes and edges are given in brackets.

	(a)	(b)	(c)	(d)	μ±σ
*d*	6.54 (4.65)	5.94 (4.55)	5.43 (4.31)	6.57 (4.65)	6.12±0.53
*R*	9.00 (6.00)	8.00 (6.00)	7.00 (5.86)	9.00 (6.00)	8.19±0.75
*D*	14.00 (9.04)	12.00 (8.90)	10.00 (8.06)	14.00 (9.06)	12.34±1.30
*C*	0.11 (0.00)	0.11 (0.00)	0.10 (0.00)	0.14 (0.00)	0.10±0.02
$\rho_{c}$	0.01 (0.13)	0.01 (0.13)	0.01 (0.13)	0.01 (0.13)	0.01±0.00

## Data Availability

The connected networks of local minima and transition states of the magnetic nanostructures discussed in this paper are available upon request.

## Abbreviations

The following abbreviations are used in this manuscript:

2D	Two-dimensional
DG	Disconnectivity graph
EL	Energy landscape
KN	Kinetic network
LM	Local minimum
TS	Transition state
MEP	Minimum-energy path
NP	Nanoparticle
MNP	Magnetic nanoparticle
RAS	Relaxation after saturation
RAQ	Relaxation after quenching
SD	Strongly-disordered
WDSL	Weakly-disordered square-lattice
L-BFGS	Limited-memory Broyden-Fletcher-Goldfarb-Shanno algorithm
